# *Streptomyces monashensis* sp. nov., a novel mangrove soil actinobacterium from East Malaysia with antioxidative potential

**DOI:** 10.1038/s41598-019-39592-6

**Published:** 2019-02-28

**Authors:** Jodi Woan-Fei Law, Hooi-Leng Ser, Nurul-Syakima Ab Mutalib, Surasak Saokaew, Acharaporn Duangjai, Tahir Mehmood Khan, Kok-Gan Chan, Bey-Hing Goh, Learn-Han Lee

**Affiliations:** 1 Novel Bacteria and Drug Discovery Research Group, Microbiome and Bioresource Research Strength, Jeffrey Cheah School of Medicine and Health Sciences, Monash University Malaysia, 47500 Bandar Sunway, Selangor Darul Ehsan Malaysia; 2 Biofunctional Molecule Exploratory Research Group, Biomedicine Research Advancement Centre, School of Pharmacy, Monash University Malaysia, 47500 Bandar Sunway, Selangor Darul Ehsan Malaysia; 3 Institute of Biomedical and Pharmaceutical Sciences, Guangdong University of Technology, Guangzhou, 510006 P. R. China; 4 UKM Medical Molecular Biology Institute (UMBI), UKM Medical Centre, University Kebangsaan Malaysia, Kuala Lumpur, Malaysia; 5 Center of Health Outcomes Research and Therapeutic Safety (Cohorts), School of Pharmaceutical Sciences, University of Phayao, Phayao, Thailand; 6 Pharmaceutical Outcomes Research Center (CPOR), Faculty of Pharmaceutical Sciences, Naresuan University, Phitsanulok, Thailand; 7 Division of Physiology, School of Medical Sciences, University of Phayao, Phayao, Thailand; 8 The Institute of Pharmaceutical Sciences, University of Veterinary and Animal Sciences, Lahore, Pakistan; 9 Division of Genetics and Molecular Biology, Institute of Biological Sciences, Faculty of Science, University of Malaya, 50603 Kuala Lumpur, Malaysia; 10 International Genome Centre, Jiangsu University, Zhenjiang, China

**Keywords:** Bacterial evolution, Bacteriology

## Abstract

A new *Streptomyces* species discovered from Sarawak mangrove soil is described, with the proposed name – *Streptomyces monashensis* sp. nov. (strain MUSC 1J^T^). Taxonomy status of MUSC 1J^T^ was determined via polyphasic approach. Phylogenetic and chemotaxonomic properties of strain MUSC 1J^T^ were in accordance with those known for genus *Streptomyces*. Based on phylogenetic analyses, the strains closely related to MUSC 1J^T^ were *Streptomyces corchorusii* DSM 40340^T^ (98.7%), *Streptomyces olivaceoviridis* NBRC 13066^T^ (98.7%), *Streptomyces canarius* NBRC 13431^T^ (98.6%) and *Streptomyces coacervatus* AS-0823^T^ (98.4%). Outcomes of DNA–DNA relatedness between strain MUSC 1J^T^ and its closely related type strains covered from 19.7 ± 2.8% to 49.1 ± 4.3%. Strain MUSC 1J^T^ has genome size of 10,254,857 bp with DNA G + C content of 71 mol%. MUSC 1J^T^ extract exhibited strong antioxidative activity up to 83.80 ± 4.80% in the SOD assay, with significant cytotoxic effect against colon cancer cell lines HCT-116 and SW480. *Streptomyces monashensis* MUSC 1J^T^ (=DSM 103626^T^ = MCCC 1K03219^T^) could potentially be a producer of novel bioactive metabolites; hence discovery of this new species may be highly significant to the biopharmaceutical industry as it could lead to development of new and useful chemo-preventive drugs.

## Introduction

Natural products play an important part in the development of drugs as they have been the source of many of the active ingredients of medicines^1^. Microbes have been extensively explored as sources for bioactive natural products due to their production of unique secondary metabolites which are required for defense and survival in harsh environments^2^. Members of the phylum *Actinobacteria* have been one of the primary sources of bioactive natural products, owing to their capability to produce abundant secondary metabolites comprising diverse chemical structures and biological activities^3^. In particular, the genus *Streptomyces* has brought upon a beneficial impact to the pharmaceutical industry by accounting for approximately 80% of the *Actinobacteria* derived natural products^3–6^.

In the early 1940 s, Professor Dr. Waksman and Professor Dr. Henrici^7^ proposed the genus *Streptomyces* comprising Gram positive filamentous bacteria that are well-known as prolific producers of numerous compounds with various bioactivities including antibacterial, antifungal, antioxidant, anticancer, and immunosuppression^5,8–10^. Thus far, the exploration of new taxa is one of the successful approaches to uncover new chemical scaffolds or therapeutic agents^11^. Interest in the beneficial properties of *Streptomyces* has led to efforts to explore these organisms found in a variety of habitats such as terrestrial, marine, desert, and plants - resulting in about 844 validly identified species to date (http://www.bacterio.cict.fr/)^12,13^. Recently, there has been increasing scientific interest in the discovery of novel *Streptomyces* from underexplored area such as the mangrove environment, in hopes that this could lead to the extraction of new and useful compounds from these novel species^14,15^. In fact, mangrove environments are currently considered one of the best marine resources for the isolation of novel *Streptomyces*^16^.

Globally, the largest percentage distribution of mangrove forests of 42% is found to be in Asia, followed by 20% in Africa, 15% in North and Central America, 12% in Oceania, and 11% South America^17^. Malaysia is among the most mangrove-rich country in Asia with the state of Sarawak being an area which has abundant mangrove forests that are mostly remained undisturbed^18^. Mangrove environments are unique and dynamic as they are mainly situated in the intertidal zones of tropical and subtropical coastal regions^19^. Furthermore, a variety of terrestrial, freshwater, and marine organisms inhabit the mangrove forests^20^. Mangroves are vastly rich in nutrient and organic matter resulting from countless microbial enzymatic and metabolic activities^21^. In addition, mangrove environments experience alterations in salinity and tidal gradient constantly. All these factors will eventually assist in the rapid development of species diversity which occurs as a reaction to environmental variations and triggers metabolic pathway adaptations in living organisms which could result in generation of imperative metabolites^3,21^. Hence, these reasons have essentially driven the investigation of *Streptomyces* population present in Sarawak mangrove forests which then created a chance for novel species discovery.

Mangrove derived *Streptomyces* are a valuable source of bioactive secondary metabolites^22^. The production of secondary metabolites by *Streptomyces* often occurs when environmental stresses are present, such as, presence of competing microorganisms or nutrient depletion^23^. Upon exposure to stressful conditions like depletion of nutrients, *Streptomyces* bacteria undergo complex morphological changes, during which they initially develop a network of branched filaments known as the substrate mycelium (vegetative phase) and subsequently form aerial multinucleated mycelium and spores (reproductive sporulation phase)^23,24^. During this shifting phase, many interesting secondary metabolites are produced to ensure the survival of *Streptomyces* under stressful or unfavorable environments^24^. Additionally, *Streptomyces* have a large genome of approximately 8–10 Mbp containing more than 20 biosynthetic gene clusters that encode enzymes for the biosynthesis of secondary metabolites^25^. Aside from ensuring the survival of the organism, this unique characteristic of *Streptomyces* hints at the capability to produce novel bioactive secondary metabolites. The bioactive secondary metabolites produced by *Streptomyces* are structurally diverse^26^; the commonly found compounds include polyketides, peptides, pyrroles, β-lactams, and terpenes^23,24^. Many novel bioactive compounds have been discovered from mangrove derived *Streptomyces* including: (1) chalcomycin B, a novel macrolide antibiotic isolated from *Streptomyces* sp. B7064^27^; (2) xiamycin A, a novel pentacyclic indolosesquiterpene with anti-HIV activity isolated from *Streptomyces* sp. GT20021503^28^; (3) bafilomycin K, a novel antifungal macrolide isolated from *Streptomyces flavotricini* Y12-26^29^; and (4) streptocarbazoles A and B, novel indolocarbazoles with cytotoxic activity isolated from *Streptomyces* sp. FMA^30^.

Also, there is increasing evidence that novel *Streptomyces* from the mangrove are valuable sources of antioxidant and anticancer compounds. A study conducted by Hong *et al*.^31^ found that new species *Streptomyces* isolate 162227 and 0614149 isolated from mangrove sites in China were capable of inhibiting Human Colon Tumor 116 cells. In Malaysia, a number of novel *Streptomyces* strains have been identified from mangrove environments. For instance, *Streptomyces pluripotens*^20^, *Streptomyces mangrovisoli*^32^, *Streptomyces humi*^33^, *Streptomyces antioxidans*^15^, *Streptomyces malaysiense*^14^, and *Streptomyces colonosanans*^5^. Some of these novel mangrove *Streptomyces* have been associated with potential antioxidant and anticancer activities, for example, *Streptomyces mangrovisoli* exhibited strong antioxidant activity and the antioxidant agent was identified as Pyrrolo[1,2-a]pyrazine-1,4-dione, hexahydro-^32^. *Streptomyces malaysiense* and *Streptomyces colonosanans* were reported to exhibit strong antioxidant activity as well as demonstrating cytotoxicity against colon cancer cell lines^5,14^.

Oxidative stress is a condition where there is a cumulative production of oxygen free radicals through either endogenous or exogenous insults along with insufficient antioxidant defense, and has been associated with carcinogenesis^8,34^. The accumulation of free radicals may cause modification or damage to vital biological macromolecules such as lipids, proteins, and DNA. As a result, DNA mutations might occur which could increase cancer risk^32,34^. Antioxidants play a vital role in biological systems by scavenging the excessive free radicals in order to prevent the harmful effects caused by oxidative stress^5^. Given that cancer is a major public health issue, scientists are actively searching for effective cancer treatment options which include the discovery of potent natural antioxidant and anticancer agents from microbial sources^5,35,36^. *Streptomyces* is proven to be a good source of anticancer drugs; a number of anticancer drugs currently in use have been derived from *Streptomyces* such as bleomycin, dactinomycin, mitomycin C, and doxorubicin^37–40^. Hence, this triggered our interest to look into the potential antioxidant and anticancer activities of Sarawak mangrove-derived *Streptomyces*.

This study was conducted to investigate novel *Streptomyces* strains isolated from mangrove soil sampled at Sarawak, East Malaysia. Strain MUSC 1J^T^ was recovered from one of the soil samples and polyphasic approach based on genotypic, chemotaxonomic and phenotypic features verified that it is a novel *Streptomyces* species. Whole genome of strain MUSC 1J^T^ was analyzed via next generation sequencing technique. This study further explored the antioxidant and cytotoxic potentials of the extract of this bacterium. With the application of gas chromatography-mass spectrometry (GC-MS), the active compounds present in the extract that were accountable for the observed bioactivities were identified. The outcome of current research provides an in depth understanding of *Streptomyces monashensis* sp. nov. MUSC 1J^T^ from different perspectives and also demonstrates the potential of this strain in producing bioactive compounds with antioxidant and cytotoxic activities.

## Results

### Genotypic, phylogenetic, and genomic analyses of strain MUSC 1J^T^

**Figure 1:**
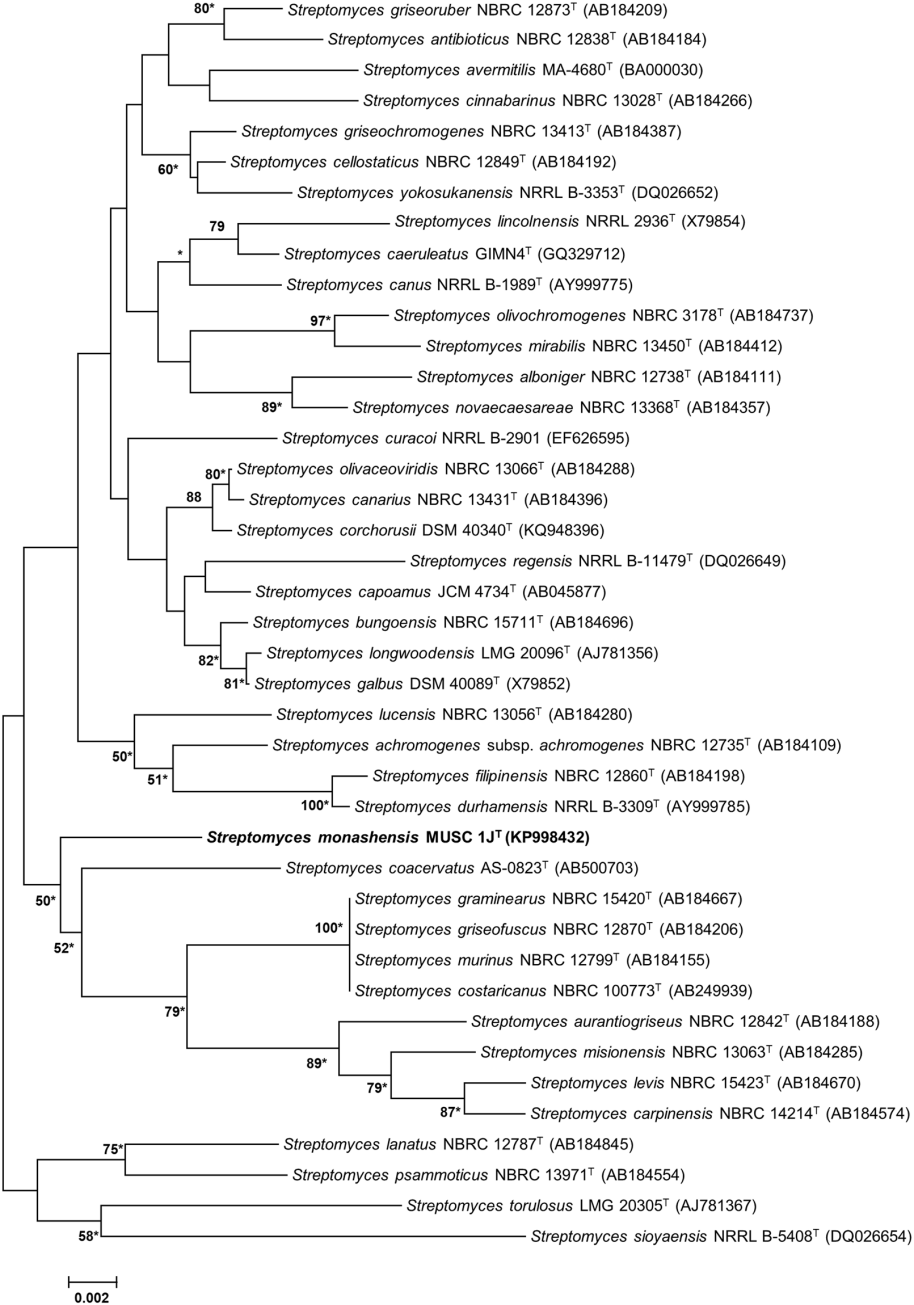
Neighbour-joining phylogenetic tree based on almost complete 16S rRNA gene sequences (1490 nucleotides) showing the relationship between *Streptomyces monashensis* MUSC 1J^T^ and representatives of some other related taxa. Numbers at nodes indicate percentages of 1000 bootstrap re-samplings, only values above 50% are shown. Bar, 0.002 substitutions per site. Asterisks indicate that the corresponding nodes were also recovered using the maximum-likelihood and maximum-parsimony tree-making algorithms.

The nearly full-length 16S rRNA gene sequence was attained for strain MUSC 1J^T^ (1490 bp; GenBank/EMBL/DDBJ accession number KP998432). Based on the 16S rRNA sequences, phylogenetic trees were reconstructed to determine the evolutionary relationship of this strain with its related type strains (Figs 1, S1 and S2). Results were in agreement that the most closely related strain is *S*. *coacervatus* AS-0823^T^ (98.4% sequence similarity) with shortest evolutionary distance, as they formed distinct clade at bootstrap value of ≥50% in the neighbour-joining (Fig. 1), maximum-likelihood (Fig. S1), and maximum-parsimony (Fig. S2) phylogenetic trees. The 16S rRNA gene sequence analysis for strain MUSC 1J^T^ revealed that this strain exhibited the highest similarity to strain *S*. *corchorusii* DSM 40340^T^ (98.7%), *S*. *olivaceoviridis* NBRC 13066^T^ (98.7%), and *S*. *canarius* NBRC 13431^T^ (98.6%).

Furthermore, the results of DDH revealed that the DNA–DNA relatedness levels between strain MUSC 1J^T^ and *S*. *corchorusii* JCM 4467^T^ (34.8 ± 3.3%), *S*. *olivaceoviridis* JCM 4499^T^ (49.1 ± 4.3%), *S*. *canarius* JCM 4549^T^ (19.7 ± 2.8%) and *S*. *coacervatus* JCM 17318^T^ (21.1 ± 3.2%) were significantly below 70%–recommended cut-off point for the delineation of bacterial species^41^. Besides, strain MUSC 1J^T^ yielded a distinctive BOX-PCR fingerprint which can be differentiated from its closely related type strains (Supplementary Fig. S3). The results of phylogenetic analysis, DDH, and BOX-PCR fingerprint analysis were consistent and thus supported that strain MUSC 1J^T^ represents a novel species of *Streptomyces* genus.

In addition, the whole genome sequencing showed that the genome of strain MUSC 1J^T^ consists of 10,254,857 bp with average coverage of 170.0-fold (Table 1). The whole project of strain MUSC 1J^T^ was deposited at DDBJ/EMBL/GenBank under accession number MLYO00000000 and the version described in this paper is the first version (MLYO0100000). A total of 9,310 coding genes was predicted on MUSC 1J^T^ genome, which assigned to 445 subsystems, along with 68 tRNA and 4 rRNA genes. Based on RAST annotation, the majority of the genes are involved in amino acid and derivative metabolism (8.06%), carbohydrate metabolism (7.45%), followed by cofactor, vitamin, prosthetic group, and pigment metabolism (4.19%).

**Table 1:** General features of *Streptomyces monashensis* MUSC 1J^T^ genome.

	*Streptomyces monashensis* MUSC 1J^T^
Genome size (bp)	10,254,857
Contigs	218
Contigs N_50_ (bp)	159,229
G + C content %	71
Protein coding genes	9,310
tRNA	68
rRNA	2 (5S), 1 (16S), 1 (23S)

**Figure 2:**
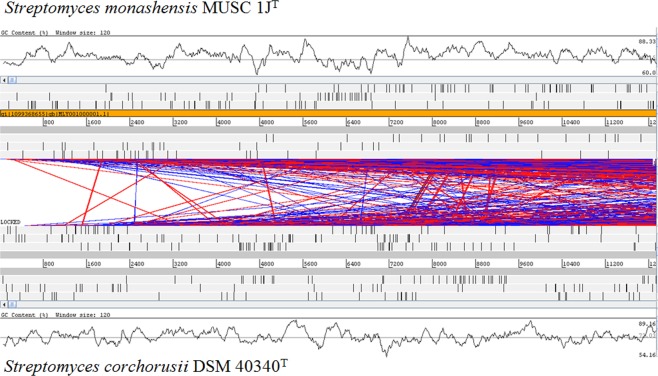
Synteny map of *Streptomyces monashensis* MUSC 1J^T^ (top) and *Streptomyces corchorusii* DSM 40340^T^ (bottom) genomes built using ACT.

Whole genome comparisons between strain MUSC 1J^T^ and its closely related type strain *S*. *corchorusii* DSM 40340^T^ was also performed. Analysis based on Clusters of Orthologous Groups (COG) functional categories showed that similar distribution of genes between strain MUSC 1J^T^ and *S*. *corchorusii* DSM 40340^T^; highest number of known proteins were found to be involved in essential processes like transcription (Class K) followed by carbohydrate transport and metabolism (Class G) (after removing uninformative classes such as R and S in the analysis) (Table 2). Further analysis using Artermis Comparison Tool (ACT)^42^ which uses BLAST to compare two or more genomes revealed large amount of synteny exists between strain MUSC 1J^T^ and *S*. *corchorusii* DSM 40340^T^ (Fig. 2). Nonetheless, the ANI value comparing strain MUSC 1J^T^ and *S*. *corchorusii* DSM 40340^T^ was calculated to be 86.03%. ANI has become increasingly popular due to the availability of whole genome sequences. The ANI analysis is primarily done by computation comparisons of two genome sequences to determine the genetic relatedness between prokaryotic strains^43^. A report by Goris *et al*.^44^ has described that 95% ANI and 69% conserved DNA corresponded with the cut-off point of 70% DDH for species delineation. The ANI value reflected by strain MUSC 1J^T^ and type strain *S*. *corchorusii* DSM 40340^T^ was found to be well below the recommended value by Goris *et al*.^44^. This finding was also in line with the outcome of DDH analysis between strain MUSC 1J^T^ and *S*. *corchorusii* DSM 40340^T^ (DNA-DNA relatedness of 34.8 ± 3.3%, <70%). Furthermore, additional analyses of strain MUSC 1J^T^ and its other closely related strains that possessed >98% 16S rRNA sequence similarity have revealed ANI values between 82–87%, which falls significantly below the recommended value (Table S1). Therefore, the novel status of the strain MUSC 1J^T^ was further confirmed based on these extensive genomic comparative analyses.

**Table 2:** Comparison between MUSC 1J^T^ and *Streptomyces corchorusii* DSM 40340^T^ based on COG functional categories.

*Class*	*MUSC 1J*^T^		*S. corchorusii*		*Description*
	Counts	%	Counts	%	
A	5	0.07	7	0.09	RNA processing and modification
B	1	0.01	1	0.01	Chromatin structure and dynamics
C	468	6.37	466	6.21	Energy production and conversion
D	58	0.79	51	0.68	Cell cycle control, cell division, chromosome partitioning
E	574	7.81	591	7.88	Amino acid transport and metabolism
F	131	1.78	125	1.67	Nucleotide transport and metabolism
G	638	8.68	649	8.65	Carbohydrate transport and metabolism
H	291	3.96	287	3.83	Coenzyme transport and metabolism
I	360	4.90	371	4.95	Lipid transport and metabolism
J	229	3.12	235	3.13	Translation, ribosomal structure and biogenesis
K	961	13.08	995	13.26	Transcription
L	252	3.43	240	3.20	Replication, recombination and repair
M	307	4.18	312	4.16	Cell wall/membrane/envelope biogenesis
N	7	0.10	11	0.15	Cell motility
O	200	2.72	201	2.68	Posttranslational modification, protein turnover, chaperones
P	270	3.67	233	3.11	Inorganic ion transport and metabolism
Q	389	5.29	358	4.77	Secondary metabolites biosynthesis, transport and catabolism
R	983	13.38	1100	14.66	General function prediction only
S	487	6.63	492	6.56	Function unknown
T	518	7.05	559	7.45	Signal transduction mechanisms
U	63	0.86	58	0.77	Intracellular trafficking, secretion, and vesicular transport
V	153	2.08	157	2.09	Defense mechanisms
W	1	0.01	1	0.01	Extracellular structures
Z	2	0.03	2	0.03	Cytoskeleton
Total	7348	100	7502	100

**Figure 3:**
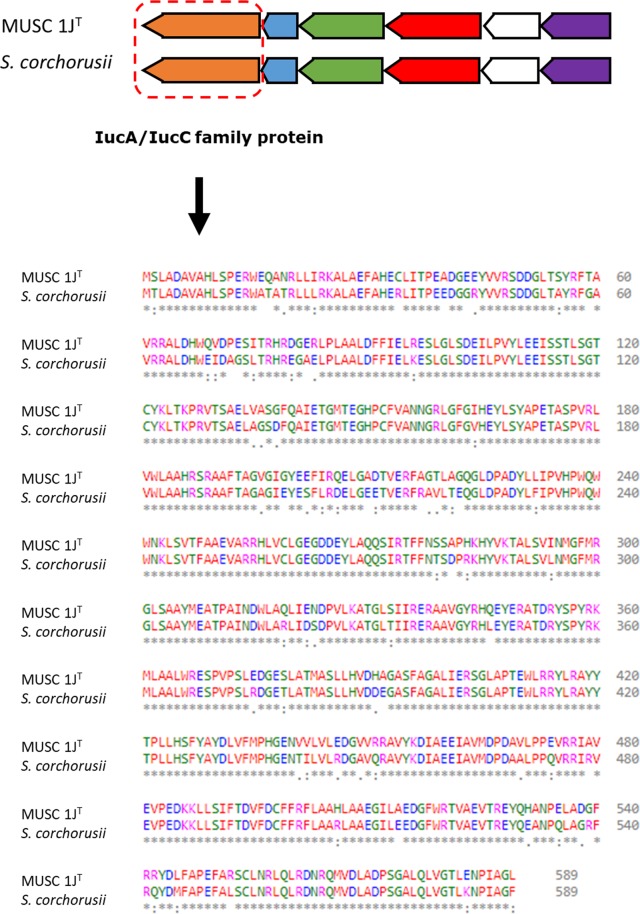
Biosynthetic gene clusters related to production of siderophore, desferrioxamine B for *Streptomyces monashensis* MUSC 1J^T^ and *Streptomyces corchorusii* DSM 40340^T^.

Apart from that, both of the genomes were also submitted to antiSMASH to detect presence of biosynthetic gene clusters. From the analysis, more than 120 clusters were detected on strain MUSC 1J^T^ genome related to various biosynthetic gene clusters including type-I polyketide synthetase, indole biosynthesis, and siderophores production. One of the common biosynthetic gene clusters within strain MUSC 1J^T^ and *S*. *corchorusii* was selected for comparison – biosynthetic gene cluster related to production of desferrioxamine B. The gene clusters were highly similar and pairwise comparison of the gene encoding for IucA/IucC family protein responsible for production of desferrioxamine revealed that gene similarities of 88.29% (Fig. 3)^45^. The presence of these biosynthetic gene clusters indicates the bioactive potential of strain MUSC 1J^T^ and suggesting its ability in producing such valuable bioactive compounds.

### Chemotaxonomic analyses of strain MUSC 1J^T^

The results of chemotaxonomic analyses revealed that strain MUSC 1J^T^ presented a type I cell-wall as it contains LL-diaminopimelic acid^46^, an amino acid found to be present in many other species of the genus *Streptomyces*^5,19,20,32,47–49^. The predominant menaquinones of strain MUSC 1J^T^ were identified as MK-9(H_8_) (55%) and MK-9(H_6_) (16%). The detection of these predominant menaquinones is in agreement with the report of Kim *et al*.^50^. The whole cell sugars detected were glucose and ribose. Strain MUSC 1J^T^ has a G + C content of 71 mol% and it was in the range of 67.0–78.0 mol% as described for *Streptomyces*^50^.

**Figure 4:**
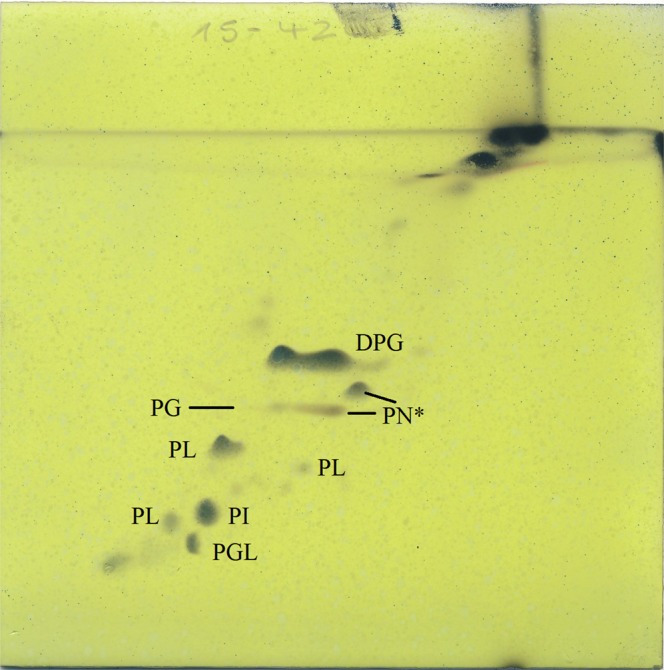
Two dimensional total lipid profile of *Streptomyces monashensis* MUSC 1J^T^. DPG, diphosphatidylglycerol; PG, phosphatidylglycerol; PGL, phosphoglycolipid; PI, phosphatidylinosotitol; PL, phospholipid; PN*, possibility of PME, phosphatidylmonomethylethanolamine/PE, phosphatidylethanolamine/OH-PE, hydroxyphosphatidylethanolamine.

The fatty acid profiles of strain MUSC 1J^T^ and its closely related type strains are presented in Table 3. The major cellular fatty acids in strain MUSC 1J^T^ were identified as anteiso-C_15: 0_ (19.3%), iso-C_16: 0_ (19.1%), iso-C_15: 0_ (13.0%), anteiso-C_17: 0_ (11.2%), and C_16: 0_ (10.8%). The fatty acid profile of strain MUSC 1J^T^ displayed high levels of similarities with those of closely related phylogenetic neighbors such as *S*. *coacervatus* JCM 17318^T^, *S*. *olivaceoviridis* JCM 4499^T^ and *S*. *corchorusii* JCM 4467^T^, as they also contain anteiso-C_15: 0_ (19.3–28.6%) as their major fatty acid (Table 3). However, quantitative differences can be observed in the fatty acid profiles of strain MUSC 1J^T^ and its closely related type strains; for example, anteiso-C_15: 0_ (19.3%) was found to be predominant in strain MUSC 1J^T^ (Table 3), but the quantity of the same fatty acid was much higher in *S*. *olivaceoviridis* JCM 4499^T^ (28.6%). Polar lipids analysis revealed the presence of phospholipid, phosphatidylglycerol, phosphatidylinositol, phosphoglycolipid, and diphosphatidylglycerol in strain MUSC 1J^T^ (Fig. 4). Outcomes of polar lipids analysis of closely related type strains were included as supplementary information (Supplementary Fig. S4).

**Table 3:** Cellular fatty acid composition of *Streptomyces monashensis* MUSC 1J^T^ and its closely related *Streptomyces* species.

Fatty acid	1	2	3	4
iso-C_12:0_	0.2	0.1	—	—
C_12:0_	0.2	0.1	—	—
iso-C_13:0_	0.4	0.1	0.2	0.2
anteiso-C_13:0_	0.5	0.2	0.3	0.2
C_13:0_	0.2	—	—	—
iso-C_14:0_	5.2	5.1	1.6	2.6
C_14:0_	0.9	0.8	0.5	0.5
iso-C_15:0_	13.0	5.1	7.9	8.4
anteiso-C_15:0_	19.3	25.0	28.6	26.2
C_15:0_	3.9	1.4	1.6	1.7
iso-C_16:1_ H	0.2	0.2	—	0.2
iso-C_16:0_	19.1	22.4	11.5	16.4
C_16:1_ Cis 9	0.9	1.7	0.4	0.4
C_16:0_	10.8	13.3	11.8	10.4
C_16:0_ 9Methyl	0.7	0.5	0.6	0.8
anteiso-C_17:1_ C	0.5	0.8	1.0	0.9
iso-C_17:0_	7.5	4.3	7.3	7.4
anteiso-C_17:0_	11.2	15.9	23.5	19.4
C_17:1_ Cis 9	—	0.2	—	0.2
C_17:0_ Cyclo	0.3	—	0.5	0.7
C_17:0_	4.1	1.8	1.6	1.6
iso-C_18:0_	0.5	0.7	0.5	0.4
iso-C_17:0_ 2OH	—	—	—	0.2
C_18:0_	0.5	0.4	—	0.4

Strains: 1, *Streptomyces monashensis* sp. nov. MUSC 1J^T^; 2, *Streptomyces coacervatus* JCM 17318^T^; 3, *Streptomyces olivaceoviridis* JCM 4499^T^; 4, *Streptomyces corchorusii* JCM 4467^T^. −, <0.1% or not detected. All data are obtained concurrently from this study.

### Phenotypic analyses of strain MUSC 1J^T^

**Figure 5:**
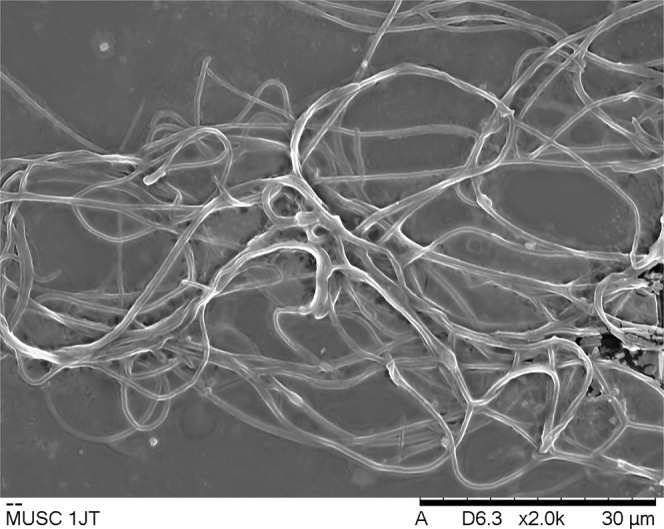
Scanning electron microscope of *Streptomyces monashensis* MUSC 1J^T^.

Phenotypic analyses in this study revealed that the mangrove forest soil-derived MUSC 1J^T^ strain grows well on ISP 2, ISP 3, ISP 5, ISP 6, ISP 7, *Streptomyces* agar, and nutrient agar after 7–14 days at 28 °C; grows moderately on starch casein agar and actinomycetes isolation agar, and does not grow on ISP 4. The colors of the aerial and substrate mycelium were media-dependent as shown in Table S2. Based on the observation of 14-day-old culture grown on ISP 2 agar, the aerial and vegetative hyphae of strain MUSC 1J^T^ were abundant and well developed. These morphological features of strain MUSC 1J^T^ (Fig. 5) conform to those observed in genus *Streptomyces*, hence, this indicated that strain MUSC 1J^T^ belongs to the genus *Streptomyces*^51^.

For the analysis of temperature, pH, and NaCl tolerance, the results indicated that growth was found to occur at 24–40 °C (optimum 28–32 °C), at pH 6.0–8.0 (optimum pH 7.0), and with 0–6% NaCl tolerance (optimum 0–2%). Cells were found to be positive for catalase and hemolytic activity. Moreover, the cells were capable of hydrolyzing soluble starch, carboxymethylcellulose, casein and tributyrin, but unable to hydrolyze chitin and xylan. In addition, the phenotypic properties presented in Table 4 demonstrated that strain MUSC 1J^T^ was distinguishable from its closely related members of the genus *Streptomyces*. The compounds listed are utilized as sole carbon sources by MUSC 1J^T^: acetic acid, α-D-glucose, α-D-lactose, α-hydroxy-butyric acid, α-keto-butyric acid, α-keto-glutaric acid, β-hydroxyl-D, L-butyric acid, β-methyl-D-glucoside, bromo-succinic acid, citric acid, D-arabitol, D-aspartic acid, D-cellobiose, dextrin, D-fructose, D-fucose, D-galactose, D-galacturonic acid, D-glucose-6-phosphate, D-gluconic acid, D-glucuronic acid, D-lactic acid methyl ester, D-malic acid, D-maltose, D-mannitol, D-mannose, D-melibiose, D-raffinose, D-saccharic acid, D-salicin, D-sorbitol, D-trehalose, D-turanose, formic acid, gelatin, gentiobiose, glucuronamide, glycerol, glycyl-L-proline, inosine, L-fucose, L-galactonic acid lactone, L-lactic acid, L-malic acid, L-rhamnose, methyl pyruvate, mucic acid, N-acetyl-β-D-mannosamine, N-acetyl-D-galactosamine, N-acetyl-D-glucosamine, pectin, p-hydroxyl-phenylacetic acid, propionic acid, quinic acid, stachyose, sucrose, Tween 40, γ-amino-butyric acid and myo-inositol. The following compounds are utilized as sole nitrogen sources by MUSC 1J^T^: L-alanine, L-arginine, L-aspartic acid, L-glutamic acid, L-histidine, L-pyroglutamic acid and L-serine. Results of chemical sensitivity assays revealed that cells are resistant to 1% sodium lactate, aztreonam, nalidixic acid, potassium tellurite, rifamycin RV and sodium bromate. While the cells are sensitive to fusidic acid, D-serine, guanine HCl, lincomycin, lithium chloride, minocycline, niaproof 4, sodium butyrate, tetrazolium blue, tetrazolium violet, troleandomycin and vancomycin.

**Table 4:** Differentiation characteristics of *Streptomyces monashensis* MUSC 1J^T^ and type strains of phylogenetically closely related species of the genus *Streptomyces*.

Characteristic	1	2	3	4
*Morphology* (*on ISP* 2):				
Color of aerial mycelium	Light Greenish Yellow	Pale Yellowish Green	Pale Yellowish Green	Yellowish White
Color of substrate mycelium	Strong Greenish Yellow	Brilliant Greenish Yellow	Pale Yellow	Pale Yellow
*Growth at:*				
26 °C	+	(+)	(+)	(+)
36 °C	(+)	+	+	+
pH 8	(+)	−	−	−
2% NaCl	+	(+)	(+)	(+)
Hemolytic	+	−	+	+
*Hydrolysis of:*				
Tributyrin (lipase)	+	+	+	−
Carboxymethylcellulose (cellulase)	+	−	+	−
*Carbon source utilization:*				
D-maltose	+	−	+	+
D-turanose	+	+	−	−
Stachyose	+	−	+	+
β-methyl-D-glucoside	+	+	−	−
D-salicin	+	+	−	−
N-acetyl-D-galactosamine	+	−	−	−
3-methyl glucose	−	+	−	−
D-fucose	+	−	+	−
D-fructose-6-PO4	−	+	+	−
D-aspartic acid	+	+	−	−
D-serine	−	−	+	−
L-galactonic acid lactone	+	−	+	+
p-hydroxy-phenylacetic acid	+	−	−	−
α-hydroxy-butyric acid	+	−	+	+
α-keto-butyric acid	+	−	+	+
acetoacetic acid	−	+	−	−
*Chemical sensitivity assays*:				
Guanidine HCl	−	+	−	−
Tetrazolium violet	−	+	−	−
Tetrazolium blue	−	+	−	−
Sodium bromate	+	+	+	−

Strains: 1, *Streptomyces monashensis* sp. nov. MUSC 1J^T^; 2, *Streptomyces coacervatus* JCM 17318^T^; 3, *Streptomyces olivaceoviridis* JCM 4499^T^; 4, *Streptomyces corchorusii* JCM 4467^T^. All data were obtained concurrently in this study. ^+^Positive; ^−^negative; ^(+)^weak.

All strains are positive for production of catalase, protease, and amylase; whilst negative for production of xylanase and chitinase.

All strains are positive for utilization of acetic acid, α-D-lactose, β-hydroxyl-D, L-butyric acid, citric acid, dextrin, D-galacturonic acid, D-gluconic acid, D-glucuronic acid, D-mannose, D-melibiose, D-raffinose, D-sorbitol, D-trehalose, gelatin, gentiobiose, glycyl-L-proline, L-fucose, L-malic acid, mucic acid, pectin, Tween 40 and γ-amino-butyric acid.

Results of genomic and phylogenetic analysis, chemotaxonomic and phenotypic analyses proven that strain MUSC 1J^T^ isolated from Sarawak mangrove soil is qualified to be assigned as a novel species in the genus *Streptomyces*, for which the name *Streptomyces monashensis* sp. nov. is proposed.

### Antioxidant activity of strain MUSC 1J^T^ extract

In this study, the antioxidant potential of novel strain MUSC 1J^T^ was evaluated using SOD activity assay, ABTS assay, and metal chelating assay. Based on the results of all the assays, the extract of strain MUSC 1J^T^ exhibited significant radical scavenging ability (Table 5). The capability of strain MUSC 1J^T^ extract to scavenge *in vitro* oxygen-derived species like superoxide anion (O_2_^⋅−^) was analyzed via SOD activity assay, which utilizes the 2-(4-iodophenyl)-3-(4-nitrophenyl)-5-(2,4-disulfophenyl)-2*H*-tetrazolium, monosodium salt (WST) reduction method. The superoxide anion radical in this assay is generated through hypoxanthine-xanthine oxidase reaction, followed by the reduction of WST to WST-1 yellow formazan by the superoxide radical^5,8,52^. Strain MUSC 1J^T^ extract possesses SOD-like activity up to 83.80 ± 4.80% by virtue of scavenging the superoxide anion radical and subsequently inhibiting the development of yellow WST-1 formazan. The extract exhibited significant SOD-like activity (*P* < 0.05) ranging from 42.41 ± 1.58% (at 0.25 mg/mL) to 83.80 ± 4.80% (at 2 mg/mL). In addition, antioxidant activity of strain MUSC 1J^T^ extract was confirmed by ABTS assay. The production of ABTS radical cation in this assay was initiated by the reaction between a strong oxidizing agent potassium persulfate with ABTS salt^53^. The extract was able to scavenge the ABTS radical generated in the assay with significant activity of 12.33 ± 3.07% at concentration of 2 mg/mL (Table 5).

**Table 5:** Radical scavenging activity of *Streptomyces monashensis* MUSC 1J^T^ evaluated using ABTS, metal chelating, and SOD assays.

Antioxidants assays	Concentration of strain MUSC 1J^T^ extract (mg/mL)	Mean ± standard error (%)
SOD	0.25	42.41 ± 1.58*
	0.50	66.55 ± 2.10*
	1.00	80.06 ± 3.38*
	2.00	83.80 ± 4.80*
ABTS	0.25	5.06 ± 1.84
	0.50	10.50 ± 1.04*
	1.00	9.42 ± 1.33*
	2.00	12.33 ± 3.07*
Metal chelating	0.25	11.82 ± 2.87*
	0.50	27.32 ± 2.90*
	1.00	44.84 ± 1.85*
	2.00	75.50 ± 1.44*

Symbol (*) indicates p < 0.05 significant difference between strain MUSC 1J^T^ extract and controls (without strain MUSC 1J^T^ extract).

The ability of strain MUSC 1J^T^ extract in exhibiting metal chelating activity further demonstrated its antioxidant potential. In a metal chelating assay, the ferrozine added can quantitatively form complexes with Fe^2+^, resulting in a formation of Fe^2+^-ferrozine complex that can be disrupted in the presence of other chelating agents^54^. The presence of strain MUSC 1J^T^ extract exhibited a significant metal chelating activity, with highest activity recorded at 75.50 ± 1.44% at 2 mg/mL concentration (Table 5). The antioxidative potential of MUSC 1J^T^ extract is emphasized through its metal chelating ability by preventing transition metals from promoting the generation of ROS^5,14^.

### Cytotoxic activity of strain MUSC 1J^T^ extract

**Figure 6:**
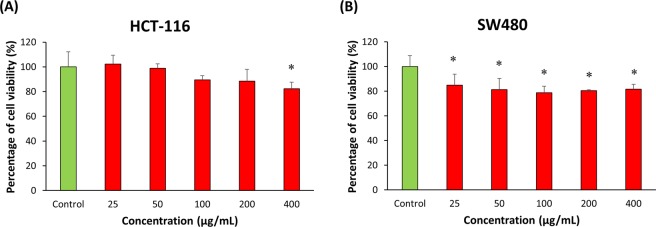
Cytotoxic activity of *Streptomyces monashensis* MUSC 1J^T^ extract against human colon cancer cell lines. The measurement of cell viability was done using MTT assay. The graphs show cytotoxicity effect of MUSC 1J^T^ extract against (**A**) SW480, and (**B**) HCT-116. All data are expressed as mean ± standard deviation and significance level are set as 0.05. Symbol (*) indicates p < 0.05 significant difference between the cells treated with MUSC 1J^T^ extract and control (without MUSC 1J^T^ extract).

Generally, strain MUSC 1J^T^ extract showed promising cytotoxic activity against the colon cancer cell lines tested. The results of strain MUSC 1J^T^ extract tested against the colon cancer cell lines were presented in Fig. 5. After 72 hours of treatment with strain MUSC 1J^T^ extract, the results revealed that the extract had significant cytotoxic effect against both colon cancer cell lines (*P* < 0.05) (Fig. 6). The extract demonstrated highest cytotoxicity against SW480, with cell viability of 81.7 ± 4.0% at the highest tested extract concentration of 400 µg/mL. As for HCT-116 colon cancer cells, the extract exhibited cell viability of 82.3 ± 5.3% at concentration of 400 µg/mL. Morphological studies were conducted using phase contrast microscopy to visualize the response of SW480 and HCT-116 cells after treated with MUSC 1J^T^ extract. It can be observed that the cancer cells have shrunk and rounded-up after treatment with MUSC 1J^T^ extract at 400 µg/mL (Supplementary Fig. S5).

### GC-MS analysis for chemical profiling of strain MUSC 1J^T^ extract

Since strain MUSC 1J^T^ exhibited significant antioxidant and cytotoxic activities in the experiments, GC-MS analysis was performed to assist in chemical profiling and the identification of compounds present in the extract. The outcome of GC-MS analysis of strain MUSC 1J^T^ extract which revealed 14 compounds is presented in Table 6: Pyrazine, 2,5-dimethyl- (**1**), Pyrazine, trimethyl- (**2**), 2-Pyrrolidone (**3**), 2-Piperidinone (**4**), Indolizine (**5**), Pyrazine, 3,5-dimethyl-2-propyl- (**6**), Phenol, 2,4-bis(1,1-dimethylethyl)- (**7**), Benzoic acid, 4-ethoxy-, ethyl ester (**8**), (3R,8aS)-3-Methyl-1,2,3,4,6,7,8,8a-octahydropyrrolo[1,2-a]pyrazine-1,4-dione (**9**), Pyrrolo[1,2-a]pyrazine-1,4-dione, hexahydro- (**10**), Phenol, 3,5-dimethoxy- (**11**), Pyrrolo[1,2-a]pyrazine-1,4-dione, hexahydro-3-(2-methylpropyl)- (**12**), 9H-Pyrido[3,4-b]indole (**13**), Pyrrolo[1,2-a]pyrazine-1,4-dione, hexahydro-3-(phenylmethyl)- (**14**), with chemical structures illustrated in Fig. 6. The main classes of compounds found in the extract include pyrazine, pyrrolidone, piperidone, indolizine, phenolic compound, benzoic acid ester, pyrrolopyrazine, and β-carboline alkaloid.

**Table 6:** Compounds identified from *Streptomyces monashensis* MUSC 1J^T^ extract using GC-MS.

No.	Retention time (min)	Compound	Class	Molecular formula	Molecular weight (MW)	Quality (%)
1	13.547	Pyrazine, 2,5-dimethyl-	Pyrazine	C_6_H_8_N_2_	108	74
2	19.675	Pyrazine, trimethyl-	Pyrazine	C_7_H_10_N_2_	122	80
3	23.869	2-Pyrrolidone	Pyrrolidone	C_4_H_7_NO	85	86
4	29.654	2-Piperidinone	Piperidone	C_5_H_9_NO	99	74
5	34.970	Indolizine	Indolizine	C_8_H_7_N	117	83
6	36.103	Pyrazine, 3,5-dimethyl-2-propyl-	Pyrazine	C_9_H_14_N_2_	150	72
7	44.485	Phenol, 2,4-bis(1,1-dimethylethyl)-	Phenolic compound	C_14_H_22_O	206	93
8	44.897	Benzoic acid, 4-ethoxy-, ethyl ester	Benzoic acid ester	C_11_H_14_O_3_	194	95
9	51.701	(3R,8aS)-3-Methyl-1,2,3,4,6,7,8,8a-octahydropyrrolo[1,2-a]pyrazine-1,4-dione	Pyrrolopyrazine	C_8_H_12_N_2_O_2_	168	90
10	53.314	Pyrrolo[1,2-a]pyrazine-1,4-dione, hexahydro-	Pyrrolopyrazine	C_7_H_10_N_2_O_2_	154	94
11	56.187	Phenol, 3,5-dimethoxy-	Phenolic compound	C_8_H_10_O_3_	154	53
12	59.523	Pyrrolo[1,2-a]pyrazine-1,4-dione, hexahydro-3-(2-methylpropyl)-	Pyrrolopyrazine	C_11_H_18_N_2_O_2_	210	78
13	60.387	9H-Pyrido[3,4-b]indole	β-carboline alkaloid	C_11_H_8_N_2_	168	95
14	72.082	Pyrrolo[1,2-a]pyrazine-1,4-dione, hexahydro-3-(phenylmethyl)-	Pyrrolopyrazine	C_14_H_16_N_2_O_2_	244	98

## Discussion

In the life cycle of *Streptomyces*, the development of aerial mycelium is initiated after 2 days and it will continue to mature into spores up to 10 days^55^. During this transition, it is when *Streptomyces* will start to produce secondary metabolites^24,55^. In this study, 10-days fermentation process was performed using a complex HFM 1 medium on strain MUSC 1J^T^ to encourage cell growth and production of secondary metabolites. The metabolites of strain MUSC 1J^T^ were then extracted using methanol as extraction solvent. The extract was subjected to bioactivity testing pertaining its antioxidant activity and cytotoxicity against cancer cells.

Oxidative stress caused by uncontrolled production of oxygen free radicals (e.g. O_2_·^−^, ·OH) has been recognized as one of the key causes of health disorders including cancer, coronary heart disease, diabetes mellitus, and neurodegenerative diseases^8,56–58^. Antioxidants can reduce the presence of free radicals, thereby protecting the human body from damage caused by oxidative stress and consequently providing a positive effect on human health by preventing or decreasing the risk of diseases such as cancer^15,57^. *Streptomyces* bacteria have been one of the high-yielding sources of natural antioxidants. Among the new antioxidants discovered from *Streptomyces* are carazostatin A isolated from *Streptomyces chromofuscus* DC 118^59^, carquinostatin A isolated from *Streptomyces exfoliates* 2419-SVT2^60^, diphenazithionin isolated from *Streptomyces griseus* ISP 5236^61^, and ageloline A isolated from *Streptomyces* sp. SBT345^62^. Results of SOD activity assay, ABTS assay, and metal chelating assay revealed the antioxidative capability of strain MUSC 1J^T^, which could suggest that the strain might be capable of producing potent antioxidant(s) that could be useful in dealing with oxidative stress.

Since the association between oxidative stress and the initiation of carcinogenesis was established, researchers have been actively searching for potential antioxidants as well as anticancer agents that could be used for prevention and/or treatment of cancer^63^. Among the different types of cancer, colorectal cancer is one of the most common cancers- ranking as the third most commonly diagnosed cancer globally and second most commonly diagnosed cancer in Malaysia^64,65^. The cytotoxic potential of strain MUSC 1J^T^ was evaluated using the MTT assay on human colon cancer cell lines: HCT-116 and SW480. Two different cancer cell lines with different genetic makeup (e.g. HCT-116 cells contain wildtype p53; SW480 cells contain mutated p53) were used as panels in this study to observe whether there is any varying efficacy in the cytotoxic activity of the extract against these cells^5,66^. As a result, slight differences in the cytotoxicity were observed in these two cancer cell lines following the exposure to strain MUSC 1J^T^ extract. This could be due to their distinctive susceptibility or resistance towards the extract which contributed by their unique genetic makeup.

**Figure 7:**
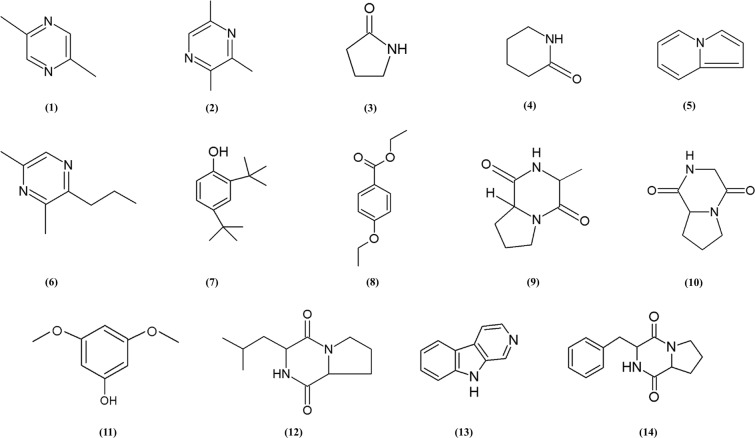
Chemical structures of constituents detected in *Streptomyces monashensis* MUSC 1J^T^ extract using GC-MS.

Further analysis such as the GC-MS analysis was performed and this allowed the identification of compounds that may account for the bioactivities exhibited by strain MUSC 1J^T^ extract. Among the identified compounds were the phenolic compounds that consist of an aromatic ring bearing one or more hydroxyl groups, also well known for their antioxidant properties^67^. The phenolic compounds detected in strain MUSC 1J^T^ extract were Phenol, 2,4-bis(1,1-dimethylethyl)- (**7**) and Phenol, 3,5-dimethoxy- (**11**) (Fig. 7). Both of the phenolic compounds were previously detected in several *Streptomyces* strains, whereby Phenol, 2,4-bis(1,1-dimethylethyl)- (**7**) in *Streptomyces cavouresis* KUV39^68^, *Streptomyces* sp. MUM256^8^, and *Streptomyces colonosanans*^5^, while both Phenol, 2,4-bis(1,1-dimethylethyl)- (**7**) and Phenol, 3,5-dimethoxy- (**11**) in *Streptomyces antioxidans*^15^. Moreover, these phenolic compounds have been associated with the antioxidant and cytotoxic activities exhibited by these *Streptomyces* strains.

Heterocyclic compounds were detected in the extract of strain MUSC 1J^T^, such instances include the pyrazines and pyrrolopyrazines. Pyrazines are heterocyclic compounds that can be found in nature and are commonly produced by microorganisms^69^. Pyrazines are typical volatile and odorous metabolites produced by *Streptomyces*^70,71^ and they have also been detected in a number of other bacteria from various sources, for instance, *Corynebacterium glutamicum*^72^, *Chondromyces crocatus*^73^, *Serratia rubidaea*, *Serratia odorifera*, *Serratia ficaria* as well as *Cedecea davisae*^74^. Some of the pyrazines were reported to be associated with antioxidant, anticancer, and antimicrobial activities^15,75,76^. Compounds Pyrazine, 2,5-dimethyl- (**1**), Pyrazine, trimethyl- (**2**), and Pyrazine, 3,5-dimethyl-2-propyl- (**6**) (Fig. 7) were previously detected in other microorganisms such as *Streptomyces citreus* CBS 109.60, *Streptomyces antioxidans*, and *Corynebacterium glutamicum*^15,70,72^. Previous studies also reported that these compounds exhibited antitumor and antioxidant activities. For example, Wang and Tao^77^ reported the detection of Pyrazine, 2,5-dimethyl- (**1**) in the metabolites of *Stigmatella* WXNXJ-B as one of the compounds contributing to the antitumor activities on human liver carcinoma cells and human breast cancer cells. As for pyrrolopyrazines, they can be found in or are produced by *Streptomyces*^32^. Pyrrolopyrazines are known to exert various bioactivities including antioxidant, antitumor, antibacterial, antifungal, and anti-angiogenesis^32,78,79^. As an example, the compound Pyrrolo[1,2-a]pyrazine-1,4-dione, hexahydro- (**10**) has been successfully purified from marine sponge-associated *Bacillus* sp. where it exhibited significant antioxidant effect which could assist in reducing oxygen free radical induced cellular oxidative damage^58^. Also, compounds (3R,8aS)-3-Methyl-1,2,3,4,6,7,8,8a-octahydropyrrolo[1,2-a]pyrazine-1,4-dione (**9**) and Pyrrolo[1,2-a]pyrazine-1,4-dione, hexahydro- (**10**) (Fig. 7) were previously detected in crude extracts of *Streptomyces pluripotens*^80^ which were suggested to be responsible for the potent antioxidant activity exerted by the strain. Additionally, pyrrolopyrazine compounds have been associated with promising anticancer activity. The findings of this study suggested that these heterocyclic compounds could have contributed to the antioxidant activity and cytotoxic activity of strain MUSC 1J^T^ extract against the tested colon cancer cells.

A tricyclic indole β-carboline alkaloid, 9H-Pyrido[3,4-b]indole (**13**) (Fig. 7) also known as norharman was detected in strain MUSC 1J^T^ extract and it has been reported to demonstrate antitumor and cytotoxic activities in previous studies^81,82^. Compound 9H-Pyrido[3,4-b]indole (**13**) was previously detected in *Pseudoalteromonas piscicida* by Zheng *et al*.^83^ and it was cytotoxic against the tested HeLa cervical-cancer cell line and the BGC-823 stomach-cancer cell line. Also, the study conducted by Tan *et al*.^8^ suggested that the presence of 9H-Pyrido[3,4-b]indole (**13**) in *Streptomyces* sp. MUM256 could be responsible for the observed anticancer effect against colon cancer cells (HCT 116, HT 29, and Caco-2).

Finally, the compounds 2-Pyrrolidone (**3**), 2-Piperidinone (**4**), Indolizine (**5**), and Benzoic acid, 4-ethoxy-, ethyl ester (**8**) (Fig. 7) discovered in the extract of strain MUSC 1J^T^ were also found in other microbes. Sathiyanarayanan *et al*.^84^ reported the detection of 2-Pyrrolidone (**3**) in *Streptomyces* sp. MAPS15 which showed antimicrobial activity. Ser *et al*.^15^ detected 2-Piperidinone (**4**) and Indolizine (**5**) in *Streptomyces antioxidans*. Benzoic acid, 4-ethoxy-, ethyl ester (**8**) was previously detected in *Bacillus* sp. and *Streptomyces colonosanans*^5,85^.

From the results of GC-MS analysis, it can be concluded that majority of the chemical compounds detected in the extract of strain MUSC 1J^T^ are recognized for their antioxidative and cytotoxic activities against cancer cells. Hence, these identified compounds might be the factors contributing to the antioxidant and cytotoxic activities demonstrated by extract from strain MUSC 1J^T^. However, additional studies are required to determine the exact compound or combination of compounds that contributed to the observed activities.

Meanwhile, the genomic studies of *Streptomyces* provide a basis for better understanding of the secondary metabolism and the production of target bioactive metabolites, thus creating an opportunity to obtain novel bioactive compounds^86,87^. With the availability of NGS technology, the whole genome of strain MUSC 1J^T^ was subjected to sequencing. The availability of whole genome sequences provides a new point of view for novel strain identification as the information allows in-depth genomic comparisons. For instance, the calculation of ANI of conserved genes present in two sequenced strains have been suggested to be comparable to results from the conventional DDH method^44,88^. Apart from that, whole genome sequences allow genome mining, which in turn enables identification of gene clusters for natural product biosynthesis, and subsequently accelerate the discovery of potential drug leads. In the current study, the biosynthetic gene clusters related to production of desferrioxamine B was detected in MUSC 1J^T^. Even though desferrioxamine has long been used clinically to treat iron toxicity, various studies suggested the potential use of this compound to manage other diseases including osteoporosis^89^, neurodegenerative diseases^90,91^ and cancer^92,93^. Nonetheless, the genome potential of MUSC 1J^T^ genome prompts application of advanced techniques like genome editing with CRISPR-Cas9 systems to accentuate its ability in producing these bioactive metabolites. Altogether, these findings highlight the value of this mangrove derived novel strain MUSC 1J^T^ in the biopharmaceutical field.

## Description of ***Streptomyces monashensis*** sp. nov

*Streptomyces monashensis* sp. nov. (mo.nash.en’sis. N.L. masc. adj. referring to Monash University).

Cells stain Gram-positive and light greenish yellow aerial and strong greenish yellow substrate mycelium on ISP 2 agar. Coloration of aerial and substrate mycelium are media-dependent (Table S2). Optimal cell growth occurred at 28–32 °C, pH 7.0, with 0–2% NaCl. Cells are positive for catalase and hemolytic activities, as well as capable of producing amylase, cellulase, protease, and lipase enzymes.

The cell wall peptidoglycan contains LL-diaminopimelic acid. The predominant menaquinones are MK-9(H_8_) and MK-9(H_6_). Whole cell sugars detected include glucose and ribose. The polar lipids consist of phospholipid, phosphatidylglycerol, phosphatidylinositol, phosphoglycolipid and diphosphatidylglycerol. The major cellular fatty acids (>10%) are anteiso-C_15: 0_, iso-C_16: 0_, iso-C_15: 0_, anteiso-C_17: 0_ and C_16: 0_.

The type strain is MUSC 1J^T^ (=DSM 103626^T^ = MCCC 1K03219^T^) isolated from mangrove sediments collected from the Sarawak mangrove forest located in East Malaysia. The 16S rRNA gene sequence of strain MUSC 1J^T^ has been deposited in GenBank/EMBL/DDBJ under the accession number KP998432. The genome size of strain MUSC 1J^T^ is 10,254,857 bp with average coverage of 170.0-fold and its G + C content is approximately 71 mol%. The whole project of strain MUSC 1J^T^ was deposited at DDBJ/EMBL/GenBank under accession number MLYO00000000 and the version described in this paper is the first version (MLYO0100000).

## Conclusion

In summary, the strain MUSC 1J^T^, a novel species of the genus *Streptomyces* was successfully isolated and identified from mangrove soil collected at the mangrove forest of Kuching, Sarawak, East Malaysia. The name *Streptomyces monashensis* sp. nov. is proposed and the type strain is MUSC 1J^T^ (=DSM 103626^T^ = MCCC 1K03219^T^). The findings of this study demonstrated that strain MUSC 1J^T^ exhibits strong antioxidant activity as high as 83.80 ± 4.80% via SOD assay as well as significant cytotoxic activity against colon cancer cell lines SW 480 and HCT-116. This study provides a comprehensive description of the novel strain *Streptomyces monashensis* MUSC 1J^T^ and elucidates the potential of the strain in the biopharmaceutical industry. The potent antioxidative activity of *Streptomyces monashensis* MUSC 1J^T^ shows the strain to be a potentially good microbial source that could contribute to drug discovery, especially with regard to development of potential antioxidant agents from this strain. Hence, it is worthwhile to conduct further studies to provide in-depth understanding on the antioxidative property of this strain.

## Materials and Methods

### Soil sampling, isolation and maintenance of strain

Soil samples were originated from a mangrove forest in Malaysia, specifically, in the area of Kuching of Sarawak. Collection of soil samples was carried out in June 2015; the isolation and maintenance of *Streptomyces* isolates were conducted according to previously described method^5^. Eighty-eight *Streptomyces* isolates were successfully recovered from the soil samples and *in vitro* preliminary bioactivity screening of methanolic *Streptomyces* extracts was performed (data not shown). Strain MUSC 1J^T^, isolated from sampling site KTTAS 5 (1°41′47.77″N 110°11′16.05″E), was discovered as one of the putative novel isolates with potential antioxidant and cytotoxic activities.

### Genotypic, phylogenetic, and genomic analyses

Methods of genomic DNA extraction of the strain were adapted from Hong *et al*.^31^ and the methods of PCR amplification of the 16S rRNA gene were adapted from Lee *et al*.^20^ using TurboCycler 2 (Blue-Ray Biotech, Taipei, Taiwan). The nearly-complete 16S rRNA gene sequence of strain MUSC 1J^T^ was obtained via molecular cloning. Multiple alignment of 16S rRNA gene sequence of strain MUSC 1J^T^ with representative sequences of related type strains in the genus *Streptomyces* was performed using CLUSTAL-X software^94^; the reference sequences were retrieved from the GenBank/EMBL/DDBJ databases. Firstly, the sequence alignment was verified manually and adjusted. Then, MEGA version 6.0^95^ was used to construct the phylogenetic trees with neighbor-joining (Fig. 1), maximum-likelihood algorithms (Supplementary Fig. S1), and maximum-parsimony algorithms (Supplementary Fig. S2). The evolutionary distances for neighbor-joining algorithm were computed by the Kimura’s two-parameter model. Tree topologies were assessed by bootstrap analyses based on 1000 resamplings method of Felsenstein^96^. The levels of sequence similarity were assessed by EzBioCloud server (http://www.ezbiocloud.net/)^97^.

Genomic DNA extraction followed by DNA-DNA hybridization (DDH)^5,14^ were performed on strain MUSC 1J^T^ and its closely related type strains *S*. *corchorusii* JCM 4467^T^, *S*. *olivaceoviridis* JCM 4499^T^, *S*. *canarius* JCM 4549^T^, and *S*. *coacervatus* JCM 17318^T^. The G + C content of strain MUSC 1J^T^ was determined and BOX-PCR fingerprinting was performed according to previously established protocol^5,98,99^.

### Chemotaxonomic characteristics

The chemotaxonomic analyses were performed by the Identification Service of the DSMZ, Braunschweig^5,14,15,19,20^, which include evaluation of: cell wall peptidoglycan, whole cell sugars, respiratory quinones, fatty acids, and polar lipids.

### Phenotypic characteristics

Cultural morphology and Gram staining of strain MUSC 1J^T^ was investigated based on established protocol^5^. ISCC-NBS color charts were used for the assignment of the colony color of strain MUSC 1J^T^. Cellular morphology of strain MUSC 1 was observed using Light microscopy (80i, Nikon) and scanning electron microscopy (JEOL-JSM 6400)^5,14^. Temperature, pH, and NaCl tolerance of strain MUSC 1JT growth were evaluated in this study^5^. Production of melanoid pigments and enzymatic activities (e.g. catalase, hemolytic, amylolytic, cellulase, lipase etc.) of strain MUSC 1J^T^ were investigated using established protocol^5,14,100^. Carbon-source utilization and chemical sensitivity of *Streptomyces* strains were analyzed using Biolog GenIII MicroPlate (Biolog, USA).

The phenotypic assays mentioned in this study were performed concurrently for strain MUSC 1J^T^, *S*. *corchorusii* JCM 4467^T^, *S*. *olivaceoviridis* JCM 4499^T^ and *S*. *coacervatus* JCM 17318^T^.

### Whole genome sequencing and bioinformatics analysis of strain MUSC 1J^T^

Genomic DNA extraction and whole genome sequencing of strain MUSC 1J^T^ were conducted according to the methods described in previous studies^5,101–107^. Trimmed sequences were *de novo* assembled with CLC Genomic Workbench version 7 (CLC bio, Denmark). Prodigal version 2.6^108^ was used for gene prediction, while RNAmmer and tRNAscan SE version 1.21 were used for rRNA and tRNA prediction^109,110^. The genome assembly was submitted to Rapid Annotation using Subsystem Technology (RAST) database and NCBI Prokaryotic Genomes Annotation Pipeline (PGAP) for annotation^5^. The genome of closely related strains (e.g. *S*. *corchorusii* DSM 40340^T^) were retrieved from NCBI database for comparison using BLAST before building synteny map using Artermis Comparison Tool (ACT)^42^. The calculations of average nucleotide identity (ANI) values were performed on EzBioCloud (https://www.ezbiocloud.net/tools/ani). AntiSMASH was used to detect presence of biosynthetic gene clusters related to secondary metabolites^111^.

### Preparation of strain MUSC 1J^T^ extract

Extract of MUSC 1JT was prepared according to previously established protocol^5,31,112^, using. HFM 1 (Biomerge, Malaysia) as fermentation medium and methanol as extracting solvent. Final extract of strain MUSC 1J^T^ was suspended in dimethyl sulphoxide (DMSO) before proceeding to bioactivity tests^5^.

### Examination of antioxidant activity of MUSC 1J^T^ extract

**Superoxide anion scavenging/superoxide dismutase (SOD)** activity the extract was investigated using SOD assay Kit–WST (Sigma-Aldrich) according to previously described protocol^5,8^. The outcome of the reaction was recorded by measuring the absorbance at 450 nm.

**The 2**,**2′-azino-bis (3-ethylbenzothiazoline-6-sulphonic acid) (ABTS) assay** was carried out for the evaluation of antioxidant activity the extract using established protocol^113^. The resultant absorbance was then measured at 743 nm; with the reduction in absorbance value as an indication of the alteration in radical amount.

**Metal Chelating** activity of the extract was investigated based on the procedure derived from earlier study^113^. Outcome of the reaction was determined through absorbance measured at 562 nm using a microplate reader.

### Maintenance and growth condition of human derived cancer cell lines

In this study, the tested human derived cancer cell lines were maintained in RPMI (Roswell Park Memorial Institute)-1640 (Gibco) supplemented with 10% fetal bovine serum and 1x antibiotic-antimycotic (Gibco) in a humidified incubator at 37 °C with 5% CO_2_ in 95% air^5,8^.

### Examination of cytotoxicity activity of MUSC 1J^T^ using 3-(4,5-dimethylthazol-2yl)-2,5-diphenyl tetrazolium-bromide (MTT) assay

This study involved the evaluation of strain MUSC 1J^T^ extract against human derived colon cancer cell lines: SW480 and HCT-116. MTT assay was used for the investigation of cytotoxic activity of strain MUSC 1J^T^ extract^8,36^. Microplate reader was used to analyze the cell viability at wavelength 570 nm (with 650 nm as reference wavelength). The morphology of the cells was observed using an inverted microscope.

### Gas chromatography-mass spectrometry (GC-MS) analysis

GC-MS analysis was conducted according to the protocol previously described by Law *et al*.^5^. The instrument involved was Agilent Technologies 6980 N (GC) equipped with 5979 Mass Selective Detector (MS), with HP-5MS (5% phenyl methyl siloxane) capillary column of dimensions 30.0 m × 250 µm × 0.25 µm and helium as carrier gas at 1 mL/min. This study utilized NIST 05 Mass Spectral Library.

### Statistical analysis

Antioxidant and cytotoxic activities assays in this study were carried out in quadruplicate. The collected data was analyzed using SPSS statistical analysis software and stated as mean ± standard deviation (SD). The significant differences between groups were determined through one-way analysis of variance (ANOVA) and appropriate post hoc test (Tukey). The significance level of *p* ≤ 0.05 was used for all data analyses in this study.

## Supplementary information

Dataset 1
