# Author Correction: *Streptomyces monashensis* sp. nov., a novel mangrove soil actinobacterium from East Malaysia with antioxidative potential

**DOI:** 10.1038/s41598-019-55964-4

**Published:** 2020-01-10

**Authors:** Jodi Woan-Fei Law, Hooi-Leng Ser, Nurul-Syakima Ab Mutalib, Surasak Saokaew, Acharaporn Duangjai, Tahir Mehmood Khan, Kok-Gan Chan, Bey-Hing Goh, Learn-Han Lee

**Affiliations:** 1 Novel Bacteria and Drug Discovery Research Group, Microbiome and Bioresource Research Strength, Jeffrey Cheah School of Medicine and Health Sciences, Monash University Malaysia, 47500 Bandar Sunway, Selangor Darul Ehsan Malaysia; 2 Biofunctional Molecule Exploratory Research Group, Biomedicine Research Advancement Centre, School of Pharmacy, Monash University Malaysia, 47500 Bandar Sunway, Selangor Darul Ehsan Malaysia; 3 Institute of Biomedical and Pharmaceutical Sciences, Guangdong University of Technology, Guangzhou, 510006 P. R. China; 4 UKM Medical Molecular Biology Institute (UMBI), UKM Medical Centre, University Kebangsaan Malaysia, Kuala Lumpur, Malaysia; 5 Center of Health Outcomes Research and Therapeutic Safety (Cohorts), School of Pharmaceutical Sciences, University of Phayao, Phayao, Thailand; 6 Pharmaceutical Outcomes Research Center (CPOR), Faculty of Pharmaceutical Sciences, Naresuan University, Phitsanulok, Thailand; 7 Division of Physiology, School of Medical Sciences, University of Phayao, Phayao, Thailand; 8 The Institute of Pharmaceutical Sciences, University of Veterinary and Animal Sciences, Lahore, Pakistan; 9 Division of Genetics and Molecular Biology, Institute of Biological Sciences, Faculty of Science, University of Malaya, 50603 Kuala Lumpur, Malaysia; 10 International Genome Centre, Jiangsu University, Zhenjiang, China

Correction to: *Scientific Reports* 10.1038/s41598-019-39592-6, published online 28 February2019

The original version of this Article contained typographical errors in the Abstract and the Main Body of the text.

In the Abstract

“*Streptomyces monashensis* MUSC 1J^T^ (=DSM 103626^T^ = MCCC 1K03221^T^) could potentially be a producer of novel bioactive metabolites; hence discovery of this new species may be highly significant to the biopharmaceutical industry as it could lead to development of new and useful chemo-preventive drugs.”

now reads:

“*Streptomyces monashensis* MUSC 1J^T^ (=DSM 103626^T^ = MCCC 1K03219^T^) could potentially be a producer of novel bioactive metabolites; hence discovery of this new species may be highly significant to the biopharmaceutical industry as it could lead to development of new and useful chemo-preventive drugs.”

Under the sub-heading Description of *Streptomyces monashensis* sp. nov

“The type strain is MUSC 1J^T^ (=DSM 103626^T^ = MCCC 1K03221^T^) isolated from mangrove sediments collected from the Sarawak mangrove forest located in East Malaysia. The 16S rRNA gene sequence of strain MUSC 1J^T^ has been deposited in GenBank/EMBL/DDBJ under the accession number KP998432.”

now reads:

“The type strain is MUSC 1J^T^ (=DSM 103626^T^ = MCCC 1K03219^T^) isolated from mangrove sediments collected from the Sarawak mangrove forest located in East Malaysia. The 16S rRNA gene sequence of strain MUSC 1J^T^ has been deposited in GenBank/EMBL/DDBJ under the accession number KP998432.”

In the Conclusion section

“The name *Streptomyces monashensis* sp. nov. is proposed and the type strain is MUSC 1J^T^ (=DSM 103626^T^ = MCCC 1K03221^T^).”

now reads:

“The name *Streptomyces monashensis* sp. nov. is proposed and the type strain is MUSC 1J^T^ (=DSM 103626^T^ = MCCC 1K03219^T^).”

This has now been corrected in the PDF and HTML versions of the Article.

