# Supplementary material for: Streptomyces monashensis sp. nov., a novel mangrove soil actinobacterium from East Malaysia with antioxidative potential
Source: Sci Rep. 2019 Feb 28;9:3056. doi: 10.1038/s41598-019-39592-6 (PMC6395624; doi:10.1038/s41598-019-39592-6)
Supplement: Supplementary file 1 — Dataset 1 [file 41598_2019_39592_MOESM1_ESM.docx]

***Streptomyces monashensis* sp. nov., a novel mangrove soil actinobacterium from East Malaysia with antioxidative potential**

**Jodi Woan-Fei Law^1,2^, Hooi-Leng Ser^1,2,3^, Nurul-Syakima Ab Mutalib^4^, Surasak Saokaew^1,5,6^, Acharaporn Duangjai^1,5,7^, Tahir Mehmood Khan^1,8^, Kok-Gan Chan^9,10*^, Bey-Hing Goh^1,2,5*^, Learn-Han Lee^1,2,5*^**

^1^Novel Bacteria and Drug Discovery Research Group (NBDD) & Biofunctional Molecule Exploratory Research Group (BMEX), Biomedicine Research Advancement Centre (BRAC), School of Pharmacy, Monash University Malaysia, 47500 Bandar Sunway, Selangor Darul Ehsan, Malaysia

^2^Biomedical Research Laboratory, Jeffrey Cheah School of Medicine and Health Sciences, Monash University Malaysia, 47500 Bandar Sunway, Selangor Darul Ehsan, Malaysia

^3^Institute of Biomedical and Pharmaceutical Sciences, Guangdong University of Technology, Guangzhou 510006, PR China

^4^UKM Medical Molecular Biology Institute (UMBI), UKM Medical Centre, University Kebangsaan Malaysia, Kuala Lumpur, Malaysia

^5^Center of Health Outcomes Research and Therapeutic Safety (Cohorts), School of Pharmaceutical Sciences, University of Phayao, Phayao, Thailand

^6^Pharmaceutical Outcomes Research Center (CPOR), Faculty of Pharmaceutical Sciences, Naresuan University, Phitsanulok, Thailand

^7^Division of Physiology, School of Medical Sciences, University of Phayao, Phayao, Thailand

^8^The Institute of Pharmaceutical Sciences, University of Veterinary and Animal Sciences, Lahore, Pakistan

^9^Division of Genetics and Molecular Biology, Institute of Biological Sciences, Faculty of Science, University of Malaya, 50603 Kuala Lumpur, Malaysia

^10^International Genome Centre, Jiangsu University, Zhenjiang, China

***Correspondence:** Learn-Han Lee (lee.learn.han@monash.edu) & Bey-Hing Goh (goh.bey.hing@monash.edu), Novel Bacteria and Drug Discovery Research Group, School of Pharmacy, Monash University, Malaysia. Kok-Gan Chan (kokgan@um.edu.my), University of Malaya, Malaysia.

## Supplementary Information


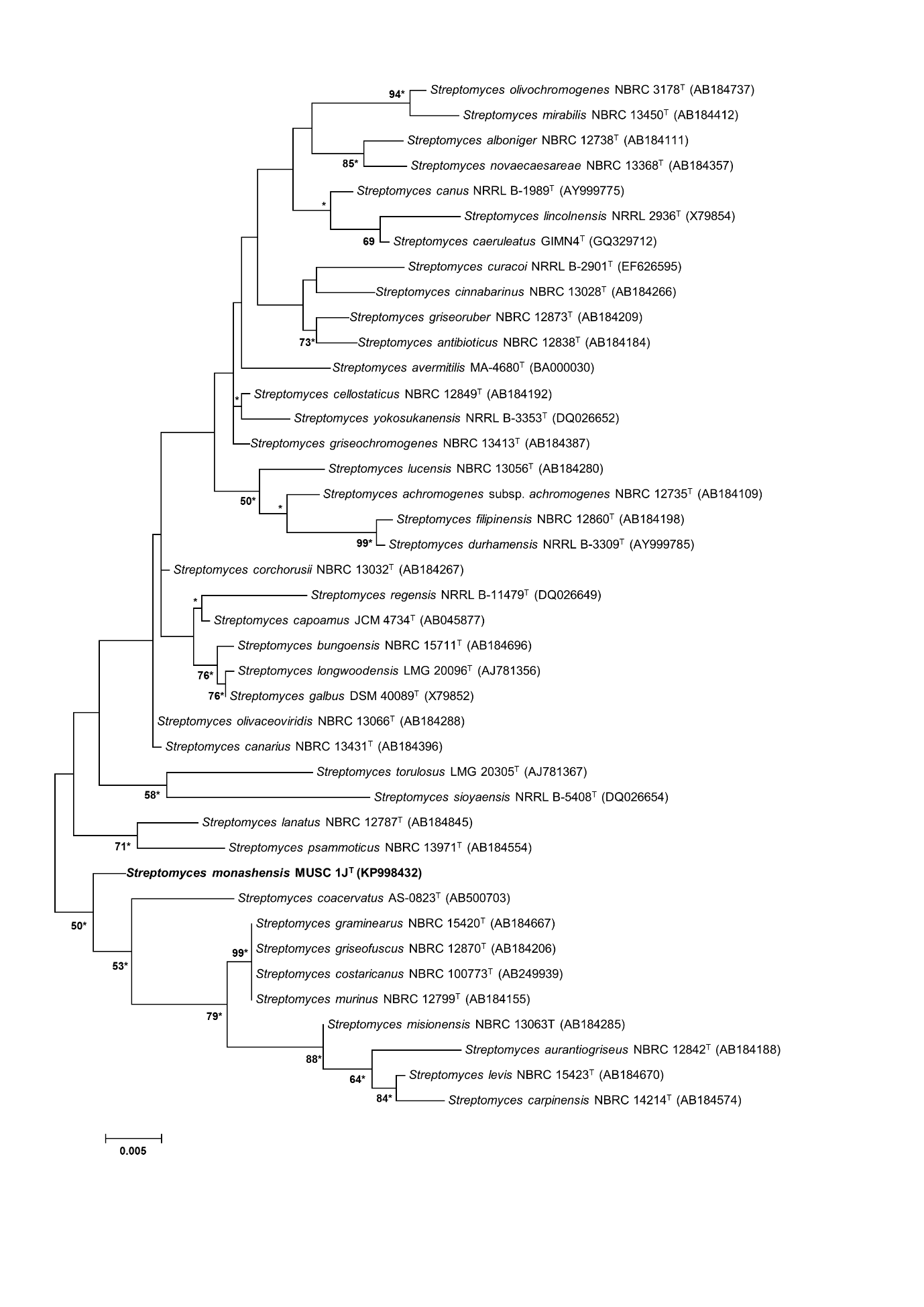


**Figure S1. Maximum-likelihood tree based on almost complete 16S rRNA gene sequences (1490 nucleotides) showing relationship between strain MUSC 1J^T^ and representatives of some other related taxa.** Bootstrap values (>50%) based on 1000 re-sampled datasets are shown at branch nodes. Bar, 0.005 substitutions per site. Asterisks indicate that the corresponding nodes were also recovered using neighbour-joining and maximum-parsimony tree-making algorithms.


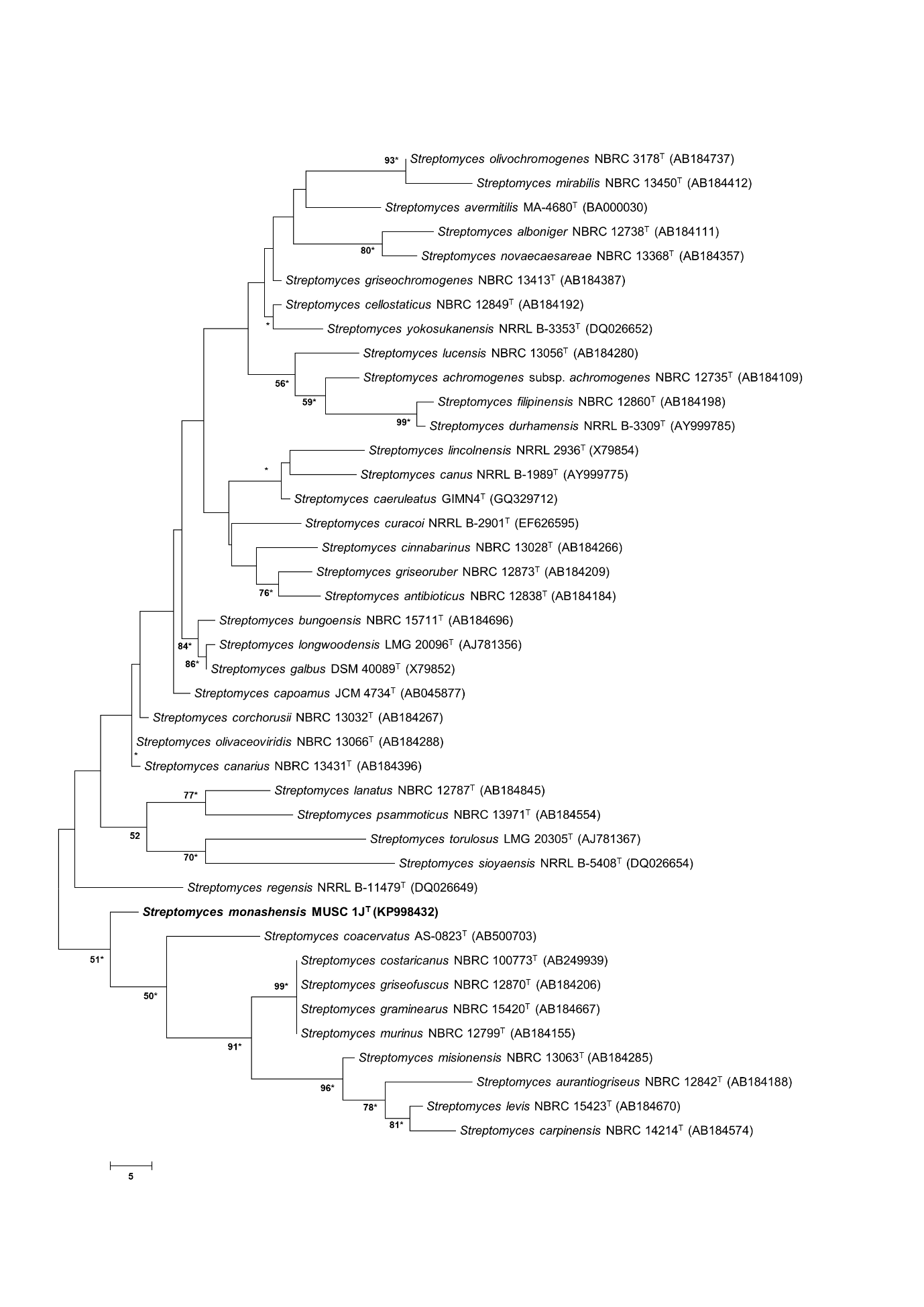


**Figure S2. Maximum-parsimony tree based on almost complete 16S rRNA gene sequences (1490 nucleotides) showing relationship between strain MUSC 1J^T^ and representatives of some other related taxa.** Bootstrap values (>50%) based on 1000 re-sampled datasets are shown at branch nodes. Bar, 5 substitutions per site. Asterisks indicate that the corresponding nodes were also recovered using neighbour-joining and maximum-likelihood tree-making algorithms.


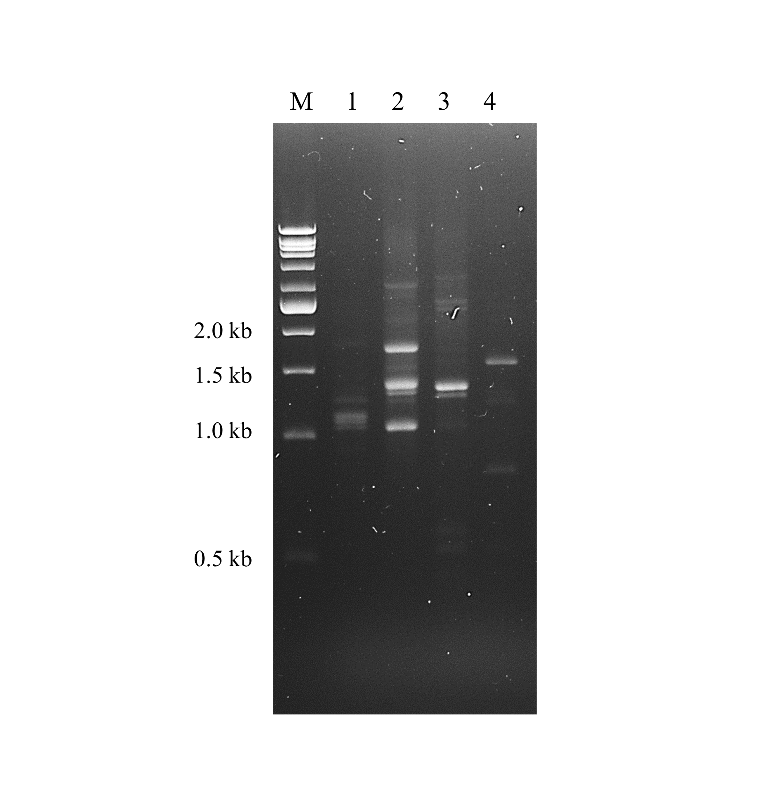


**Figure S3. BOX-PCR comparison of strain MUSC 1J^T^ and the closest related type strains.**

Lanes: 1, *Streptomyces monashensis* sp. nov. MUSC 1J^T^; 2, *Streptomyces coacervatus* JCM 17318T; 3, *Streptomyces olivaceoviridis* JCM 4499^T^; 4, *Streptomyces corchorusii* JCM 4467^T^; M, GeneRuler 1kb DNA ladder marker.


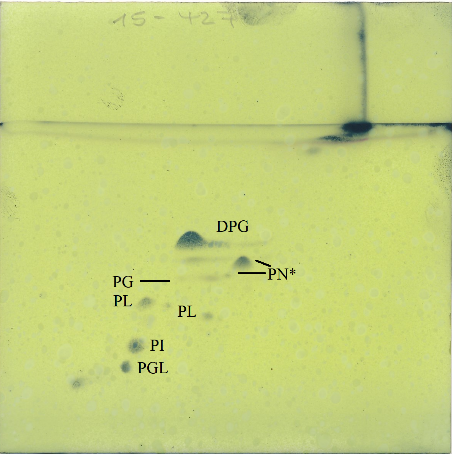

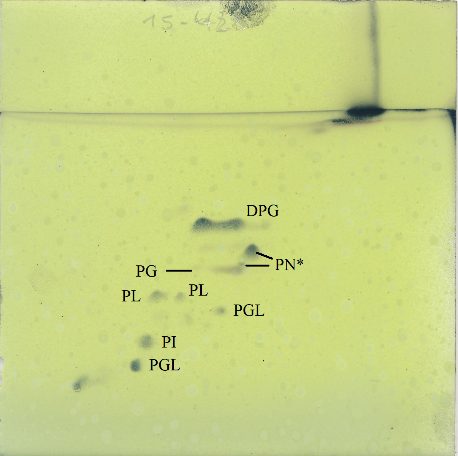


**(b)**

**(a)**

***Streptomyces coacervatus* JCM 17318^T^**

***Streptomyces olivaceoviridis* JCM 4499^T^**


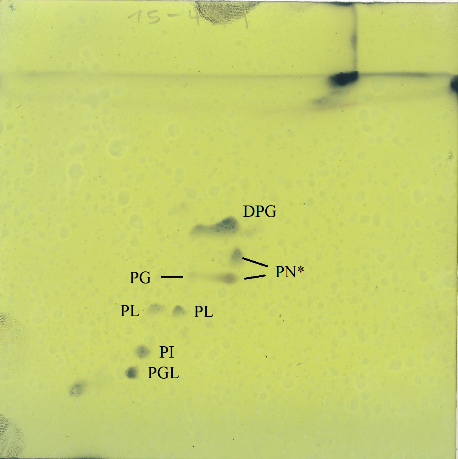


**(c)**

***Streptomyces corchorusii* JCM 4467^T^**

**Figure S4. Two dimensional total lipid profile of MUSC 1J^T^ closely related type strains: (a) *Streptomyces coacervatus* JCM 17318^T^, (b) *Streptomyces olivaceoviridis* JCM 4499^T^, and (c) *Streptomyces corchorusii* JCM 4467^T^.**

DPG, diphosphatidylglycerol; PG, phosphatidylglycerol; PGL, phosphoglycolipid; PI, phosphatidylinosotitol; PL, phospholipid; PN*, possibility of PME, phosphatidylmonomethylethanolamine/ PE, phosphatidylethanolamine)/ OH-PE, hydroxyphosphatidylethanolamine.


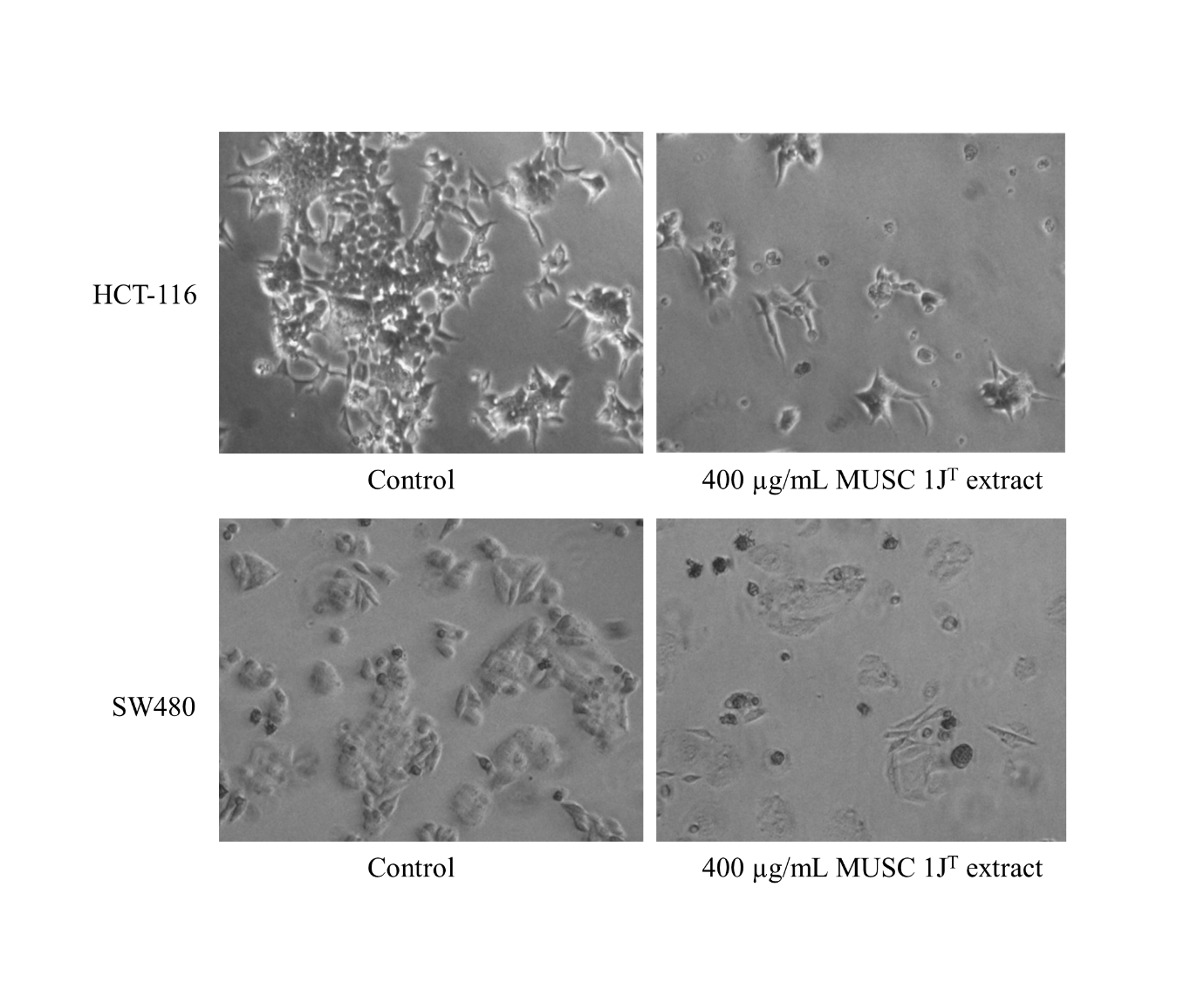


**Figure S5. Phase contrast micrograph of human colon cancer cell lines HCT-116 and SW480.** After treatment with strain MUSC 1J^T^ extract at highest concentration of 400 µg/mL, both cell lines demonstrated morphological changes where the cells appeared to be shrunken with condensed cytoplasm and detached from surface.

**Table S1. Comparison of ANI values between strain MUSC 1J^T^ and closely related *Streptomyces* strains.**

| **Species** | **Strain** | **Pairwise Similarity (%)** | **Contigs** | **Total length (bp)** | **GC (%)** | **OrthoANIu value** |
| --- | --- | --- | --- | --- | --- | --- |
| *Streptomyces corchorusii* | DSM 40340 | 98.69 | 116 | 10,285,585 | 72.03 | 86.03 |
| *Streptomyces cellostaticus* | DSM 40189 | 98.41 | 129 | 9,835,283 | 70.98 | 85.78 |
| *Streptomyces griseochromogenes* | ATCC 14511 | 98.41 | 1 | 10,764,674 | 70.76 | 85.56 |
| *Streptomyces longwoodensis* | DSM 41677 | 98.34 | 32 | 8,363,623 | 73.06 | 82.49 |
| *Streptomyces bungoensis* | DSM 41781 | 98.27 | 73 | 8,408,982 | 72.64 | 85.12 |
| *Streptomyces griseofuscus* | NBRC 12870 | 98.20 | 244 | 8,945,636 | 71.7 | 84.54 |
| *Streptomyces regensis* | NRRL B-11479 | 98.17 | 960 | 15,092,927 | 71.23 | 85.92 |
| *Streptomyces yokosukanensis* | DSM 40224 | 98.07 | 98 | 10,159,272 | 71.25 | 87.08 |
| *Streptomyces lucensis* | NBRC 13056 | 98.04 | 3,223 | 3,972,043 | 68.9 | 87.08 |

**Table S2. Cultural characteristics of strain MUSC 1J^T^ on different media at 28 ⁰C after 7-14 days of incubation.**

-, Not detected

| **Medium** | **Growth** | **Colony color** | |
| --- | --- | --- | --- |
|  |  | **Aerial mycelium** | **Substrate mycelium** |
| Yeast malt agar (ISP 2) | Good | Light greenish yellow | Strong greenish yellow |
| Oat Meal agar (ISP 3) | Good | Yellowish White | Pale yellow |
| Inorganic Salt Starch agar (ISP 4) | No growth | - | - |
| Glycerol Asparagine Agar Base (ISP 5) | Good | Pale greenish yellow | Pale yellow |
| Peptone Yeast Extract Iron agar (ISP 6) | Good | Pale yellow | Dark greenish yellow |
| Tyrosine agar base (ISP 7) | Good | Yellowish white | Pale yellow |
| *Streptomyces* agar | Good | Pale yellow | Strong yellow |
| Starch casein agar | Moderate | Greenish white | Yellowish white |
| Actinomycete isolation agar | Moderate | Yellowish white | Pale yellowish green |
| Nutrient agar | Good | Pale yellow | Brilliant greenish yellow |
